# Genome-Wide Association Study on Reproductive Traits Using Imputation-Based Whole-Genome Sequence Data in Yorkshire Pigs

**DOI:** 10.3390/genes14040861

**Published:** 2023-04-02

**Authors:** Jingchun Sun, Jinhong Xiao, Yifan Jiang, Yaxin Wang, Minghao Cao, Jialin Wei, Taiyong Yu, Xiangdong Ding, Gongshe Yang

**Affiliations:** 1 Key Laboratory of Animal Genetics, Breeding and Reproduction of Shaanxi Province, Laboratory of Animal Fat Deposition & Muscle Development, College of Animal Science and Technology, Northwest A&F University, Xianyang 712100, China; sunjc7497@nwafu.edu.cn (J.S.);; 2 National Engineering Laboratory for Animal Breeding, Laboratory of Animal Genetics, Breeding and Reproduction, Ministry of Agriculture, College of Animal Science and Technology, China Agricultural University, Beijing 100193, China

**Keywords:** genome-wide association analysis, reproductive traits, genotype imputation, Yorkshire pig

## Abstract

Reproductive traits have a key impact on production efficiency in the pig industry. It is necessary to identify the genetic structure of potential genes that influence reproductive traits. In this study, a genome-wide association study (GWAS) based on chip and imputed data of five reproductive traits, namely, total number born (TNB), number born alive (NBA), litter birth weight (LBW), gestation length (GL), and number of weaned (NW), was performed in Yorkshire pigs. In total, 272 of 2844 pigs with reproductive records were genotyped using KPS Porcine Breeding SNP Chips, and then chip data were imputed to sequencing data using two online software programs: the Pig Haplotype Reference Panel (PHARP v2) and Swine Imputation Server (SWIM 1.0). After quality control, we performed GWAS based on chip data and the two different imputation databases by using fixed and random model circulating probability unification (FarmCPU) models. We discovered 71 genome-wide significant SNPs and 25 potential candidate genes (e.g., *SMAD4*, *RPS6KA2*, *CAMK2A*, *NDST1*, and *ADCY5*). Functional enrichment analysis revealed that these genes are mainly enriched in the calcium signaling pathway, ovarian steroidogenesis, and GnRH signaling pathways. In conclusion, our results help to clarify the genetic basis of porcine reproductive traits and provide molecular markers for genomic selection in pig breeding.

## 1. Introduction

The reproductive performance of pigs plays a key role in the pig industry. Improving the reproductive performance of sows can lead to higher economic benefits for pig farms. However, reproductive traits are low-heritability traits, and their genetic structure is much more complex [1]. Therefore, it is difficult to improve these traits more rapidly using traditional breeding methods. With the development of molecular breeding technology, marker-assisted selection (MAS) and genomic selection (GS) have become effective ways to improve pig breeding efficiency [2].

In recent years, to complete genomic screening for trait-associated variants, genome-wide association studies (GWASs) have been widely applied to find quantitative trait loci (QTL) in economic traits [3]. Thus far, 35,384 QTLs have been identified in pigs according to pigQTLdb, of which 3315 QTLs are associated with reproduction (https://www.animalgenome.org/cgi-bin/QTLdb/SS/summary, 25 April 2022). In pigs, GWAS has identified numerous SNPs significantly associated with growth traits [4,5], meat quality [6,7], feed efficiency [8,9], semen traits [10,11], coat color [12,13], genetic defects [14,15], disease susceptibility [16,17], and microbial phenotypes [18]. However, most of them were genotyped based on SNP microarrays, and the density of markers is a key factor affecting GWAS efficiency [19]. With the development of sequencing technology and its increasingly low cost, many researchers have used sequencing or resequencing to perform relevant studies [20,21,22]. However, the sequencing or resequencing of large population samples is too costly and remains an inefficient strategy. Genotype imputation is an effective strategy in GWAS [23], which has been widely used in human genetics research, such as HapMap [24] and the 1000 Genomes Project [25]. It can increase the total number and density of SNPs used for association analysis and provide the opportunity to discover new potential genes.

In our study, we performed GWAS using two different genotype imputation databases and identified genetic variants related to five reproductive traits in large white pigs.

## 2. Materials and Methods

### 2.1. Ethics Statement

All ear tissue sample collection procedures were approved by the Institutional Animal Care and Use Committee of the Northwest A & F University (approval number: NWAFU-314021167).

### 2.2. Animals and Phenotypes

The pig population was uniformly reared at the core breeding farm of Zhumei Group Limited (Zhumadian City, China). Briefly, we collected breeding information and lineage records of large white pigs from 2011 to 2019 at this farm. There were 3733 pigs with complete pedigrees, and pedigrees could be traced back three generations. A total of 10,206 reproduction records of 2844 pigs were collected. The phenotype records included parity (including 8 levels: 1, 2, 3, 4, 5, 6, 7, or 8 or higher parity number), herd-year-season, and five reproductive traits. Five reproductive phenotypes, namely, total number born (TNB), number born alive (NBA), litter birth weight (LBW), gestation length (GL), and number of weaned (NW), were chosen for the next analysis. Table 1 presents the descriptive statistics of the five traits. Apart from GL, the other four traits had coefficients of variation above 25%.

### 2.3. Genotyping and Genotype Imputation

In this study, KPS Porcine Breeding 50K Chip v1 (Compass Biotechnology, Beijing, China), which contains 51,315 SNPs, was used to genotype 272 individuals of the total 2844 pigs with phenotype records. Then, quality control was performed by only keeping SNPs with MAF > 0.05, SNP call rate > 95%, individual call rate > 95%, and HWE > 1 × 10^−6^ using the PLINK software (v1.90) [26]. A total of 31,174 SNPs and 271 animals were retained for further GWAS. To improve the marker density, imputation was performed using two online software programs: the Pig Haplotype Reference Panel (PHARP v2) (http://alphaindex.zju.edu.cn/PHARP/index.php/, accessed on 21 October 2022) [27] and Swine Imputation Server (SWIM 1.0) (https://quantgenet.msu.edu/swim/index.html, 21 October 2022) [28]. After imputation with PHARP v2, quality control (R^2^ > 0.8 and MAF > 0.05) was performed, and 9,093,720 SNPs were obtained. Additionally, the SNPs were further pruned by using the “--indep-pairwise 50 5 0.9” command with a sliding window of 50 SNPs, a 5-step SNP shift, and an r^2^ less than 0.9. Similarly, SNPs imputed using the SWIM online software were subjected to the relevant quality control procedure. Finally, with the online imputation software PHARP and SWIM, 1,017,199 and 1,019,225 autosomal SNPs were retained.

### 2.4. Estimation of Genetic Parameters and Genetic Correlation

The variance and covariance components and genetic correlations of the five traits were calculated using a repeatability model in DMU v6.0 software [29].

The animal model was as follows:

(1) $$y=Xb+Z_{a}a+Z_{pe}pe+e$$

In the model, ***y*** is a vector of phenotype records; ***b*** is the fixed effect of herd-year-season and parity with eight levels; ***X*** is a design matrix relating ***b*** to ***y***; ***a*** is a vector of additive genetic effects; ***pe*** is a vector of random permanent environmental effects; and ***e*** is a vector of random residual effects. ***Z_a_*** and ***Z_pe_*** are the corresponding incidence matrices.

The genetic correlation was calculated as follows:

(2) $$r_{12}=\frac{\mathrm{cov}(a_{1},a_{2})}{\sigma_{a_{1}}\sigma_{a_{2}}}$$

where $r_{12}$ is the genetic correlation between trait 1 and trait 2, $a_{1}$ and $a_{2}$ represent the additive genetic values of trait 1 and trait 2 for the same individuals, and $\mathrm{cov}(a_{1},a_{2})$, $\sigma_{a_{1}}$, and $\sigma_{a_{2}}$ refer to the genetic covariance of two traits and the genetic standard deviations of trait 1 and trait 2, respectively.

### 2.5. Genome-Wide Association Study (GWAS)

To perform GWAS, we used the sum of an individual’s estimated breeding value (EBV) and residual as the adjusted phenotype in this study. We used fixed and random model circulating probability unification (FarmCPU) models for GWAS in GAPIT3 [30]. This method iteratively takes advantage of the mixed linear model (MLM) as the random model and stepwise regression as the fixed model [31]. In this study, we used the Bonferroni correction method to find candidate SNPs. *p* < 1/N represents the genome-wide suggestive significance threshold. *p* < 0.05/N represents the genome-wide significance threshold. Manhattan and Q-Q plots were generated using the R CMplot package version 4.2.0 [32].

### 2.6. Candidate Gene Search

We used BedTools [33] to search for candidate genes in the regions 0.5 Mb downstream and upstream of the significant SNPs based on the pig reference genome (http://useast.ensembl.org/Sus_scrofa/Info/Index/, accessed on 16 December 2022, Sscrofa11.1). Additionally, to better understand the biological processes and pathways of these candidate genes, we also performed enrichment analyses. Kyoto Encyclopedia of Genes and Genomes (KEGG) pathways and Gene Ontology (GO) terms were enriched via KOBAS-i [34].

## 3. Results

### 3.1. Genetic Parameters and Genetic Correlations of Reproductive Traits

The genetic parameters of the five reproductive traits are presented in Table 2. The heritability estimates of TNB, NBA, LBW, GL, and NW were 0.0442 ± 0.0011, 0.0442 ± 0.0012, 0.0476 ± 0.0025, 0.1571 ± 0.0009, and 0.0727 ± 0.0021, respectively. As can be seen, these traits are all low-heritability traits. Table 3 shows the genetic correlations of the five reproductive traits. The genetic correlations ranged from −0.235 to 0.985, with standard errors ranging from 0.001 to 0.015. Among the five reproductive traits, TNB, NBA, LBW, and NW show strong positive correlations, with correlations ranging from 0.751 to 0.985. In contrast, GL shows some negative correlations with the remaining four traits, but the correlations are not strong.

### 3.2. Identification of Significant SNPs Associated with Reproductive Traits before Imputation

**Figure 1:**
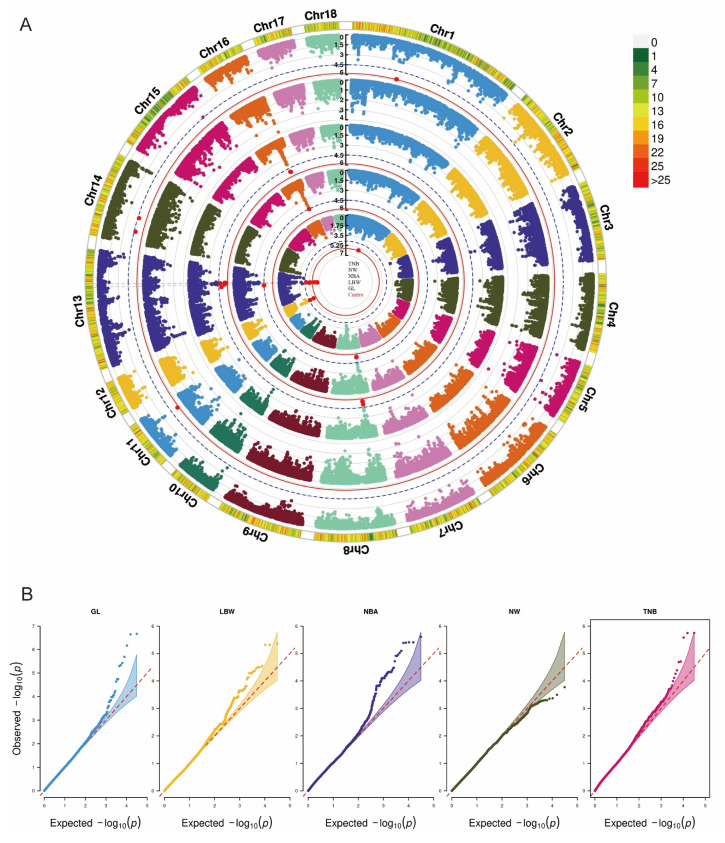
Manhattan and Q-Q plots of GWAS based on chip data for five reproductive traits. (**A**). The red line represents the genome-wide significance level. The blue line represents the suggestive significance (3.01 × 10^−5^). Red spots identify SNPs with genome-wide significance (1.60 × 10^−6^). Traits from the inner to outer lanes are gestation length (GL), litter birth weight (LBW), number born alive (NBA), number of weaned (NW), and total number born (TNB). (**B**). Q-Q plots of five reproductive traits.

In GWAS based on chip data, only three SNPs on chromosome 13 for GL reach chromosome-level significance [*p* < 1.60 × 10^−6^ (0.05/33,175)] (Figure 1A). These SNPs are located in candidate genes such as *DDPA4* and *DDPA2* (Table 4). Additionally, there are 5, 6, 11, and 7 SNPs that exceed the suggestive significance threshold [*p* < 3.01 × 10^−5^ (1/33,175)] for TNB, LBW, NBA, and GL, respectively (Supplementary Table S2).

### 3.3. Identification of Significant SNPs Associated with Reproductive Traits after Imputation with PHARP

**Figure 2:**
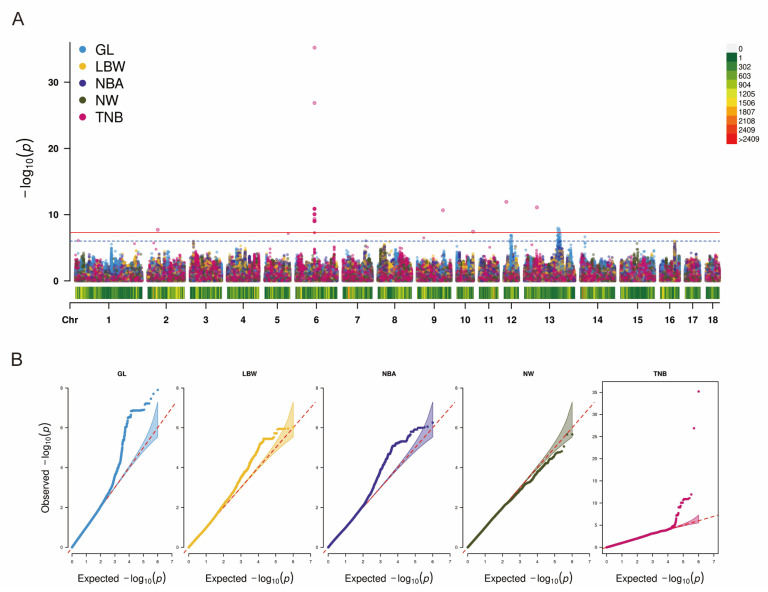
Manhattan and Q-Q plots of GWAS based on data imputation using PHARP for five reproductive traits. (**A**) The red line represents the genome-wide significance level (4.91 × 10^−8^). The blue line represents the suggestive significance (9.83 × 10^−7^). (**B**) Q-Q plots of five reproductive traits. Abbreviations: GL = gestation length, LBW = litter birth weight, NBA = number born alive, NW = number of weaned, TNB = total number born.

Figure 2A shows the results of Manhattan plots after imputation using PHARP. The Q-Q plots are shown in Figure 2B, with genome inflation factors between 0.964 and 1.095 (Supplementary Table S1). The results show that 22 and 3 genome-wide significant SNPs [*p* < 4.91 × 10^−8^ (0.05/1,017,199)] for TNB and GL are identified, respectively (Table 5). Notably, 14 genes are identified as related to reproduction, including *MRTO4*, *TAS1R2*, *PAX7*, *CAPZB*, *UBR4*, *KCNJ2*, *MITF*, *LDHA*, *LDHC*, *ABCC8*, *ARGFX*, and *IGSF11*. According to the suggestive significance threshold [*p* < 9.83 × 10^−7^ (1/1,017,199)], 8, 3, 1, and 186 SNPs are found to be associated with TNB, NBA, LBW, and GL, respectively (Supplementary Table S3).

### 3.4. Identification of Significant SNPs Associated with Reproductive Traits after Imputation with SWIM

**Figure 3:**
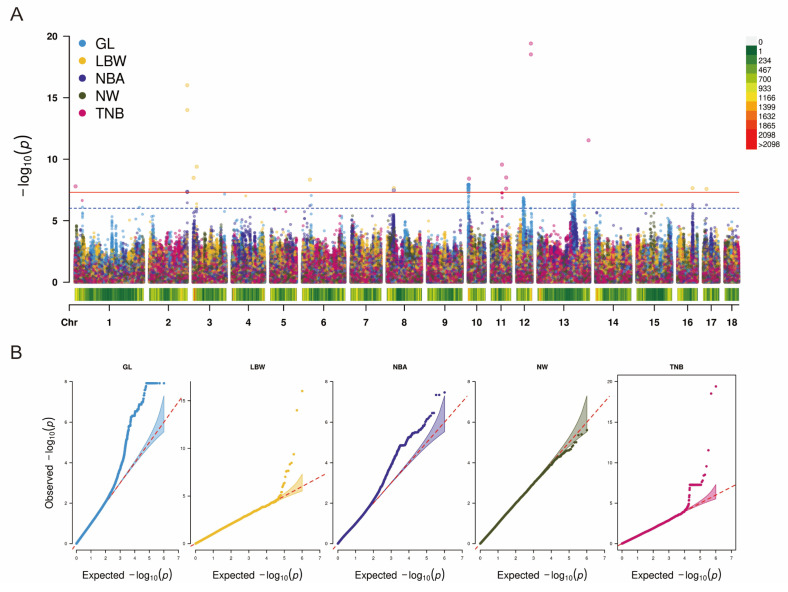
Manhattan and Q-Q plots of GWAS based on data imputation with SWIM for five reproductive traits. (**A**) The red line represents the genome-wide significance level (4.90 × 10^−8^). The blue line represents the suggestive significance (9.81 × 10^−7^). (**B**) Q-Q plots of five reproductive traits. Abbreviations: GL = gestation length, LBW = litter birth weight, NBA = number born alive, NW = number of weaned, TNB = total number born.

Figure 3A shows the Manhattan plots of the genotype imputed using SWIM for GWAS. The Q-Q plots are shown in Figure 3B, with genome inflation factors between 0.889 and 1.132 (Supplementary Table S1). Overall, 271 SNPs reaching suggestive significance [*p* < 9.81 × 10^−7^ (1/1,019,225)] were found to be associated with the five reproductive traits (Supplementary Table S4). Further, 43 SNPs reach the genome-wide significance level: 8 SNPs for TNB, 3 SNPs for NBA, 8 SNPs for LBW, and 24 SNPs for GL. These significant SNPs were annotated to a number of candidate genes associated with reproduction, such as *MYOCD*, *HMGN1*, *DACH1*, *GPC5*, *RPS6KA2*, *ARAP2*, *CAMK2A*, and *RGS18* (Table 6).

### 3.5. Bioinformatics Annotation Analysis

In this research, candidate functional genes were found by searching 0.5 Mb upstream and downstream of the suggestive SNPs using GWAS based on chip data and two imputed databases. The genes associated with TNB are found to be linked to glycolysis/gluconeogenesis, TGF-β, the oxytocin signaling pathway, and oocyte maturation processes. For NBA and LBW, the same genes, *PDGFRB*, *CAMK2A*, and *MMP2*, are identified, mainly enriched in the calcium signaling pathway, GnRH signaling pathway, and embryonic organ development process. Finally, the functional genes related to GL are enriched in the mTOR signaling pathway, ovarian steroidogenesis, prolactin signaling pathway, embryo development, and regulation of G protein-coupled receptor signaling pathway (Table 7).

## 4. Discussion

Reproductive traits such as TNB, NBA, LBW, GL, and NW are closely related to sow fertility and are important quantitative indicators of pig production. However, most of them have low heritability due to the complexity of the genetic structure. Therefore, it is important to clarify the genetic relationships between reproductive traits and to identify key candidate genes. In this study, a repeatability model was used to estimate the heritability of reproductive traits. The heritability estimates of the TNB, NBA, LBW, GL, and NW traits were 0.0442, 0.0442, 0.0476, 0.1571, and 0.0727, respectively. This is in agreement with the results of previous studies [35,36,37]. Additionally, we also calculated genetic correlations between individual traits and found strong positive correlations between TNB, NBA, LBW, and NW, with correlation coefficients ranging from 0.751 to 0.985, in agreement with previous reports [38,39]. This suggests that fewer traits can be selected to simplify breeding work.

Genotype imputation has been widely used in recent years with the development of sequencing technologies, price reductions, and the demand for high-density markers. This approach allows the imputation of chip data with low-density markers to WGS data, and the imputation accuracy is affected by the density of the target SNPs, the size of the reference population, the genetic distance between the target and imputation reference population, and the imputation procedure [40]. In our study, we imputed chip data using two publicly available online populating platforms. PHARP v2 provides genotype imputation using Minimac4, and the reference panel includes 4096 haplotypes, 53 million autosome SNPs, and 122 pig breeds [27]. The reference panel of SWIM 1.0 has a total of 2259 pigs, representing 44 different breeds. Based on the imputed data of the two imputation platforms mentioned above, combined with chip data, we performed GWAS for five reproductive traits.

In our study, we conducted GWAS for five reproductive traits using imputation data from two different online imputation platforms. Imputation data based on the SWIM platform detected more significant or potentially significant loci compared to the PHARP platform. This may be due to the fact that the SWIM platform has a larger number of pigs in its reference panel. In addition, an imputation strategy could improve on previous SNP-based studies without the need for additional data and expense. Furthermore, a common set of SNPs can be obtained with an imputation approach, thus making a meta-analysis possible.

Some studies have shown that the FarmCPU model can be effective in GWASs for identifying loci with low-heritability traits [35]. So, we performed GWAS by using the FarmCPU model, which divides the MLM into two parts and uses them iteratively [31]. For the TNB trait, a total of 19 suggestive candidate genes were identified based on chip data and imputed data. Among them, the *RPS6KA2* gene plays a major role in the EGF signaling cascade at ovulation, which is also correlated with oocyte developmental quality [41]. As a transcription factor, *SMAD4* plays an important role in the porcine reproductive system. It has been shown that miR-143 [42], miR-26b [43], and miR-10b [44] can inhibit apoptosis and promote E2 release via *SMAD4* in porcine granulosa cells. For both the NBA and LBW traits, GWAS based on imputed data identified the *CAMK2A*, *NDST1*, and *RPS14* genes. In a meta-analysis of reproductive traits in heifers, the *CAMK2A* gene was identified as being involved in calcium signaling mechanisms and acting on pituitary gonadotropin secretion [45]. This is consistent with our findings. In addition, *NDST1* has been shown to be critical for many organogenesis processes, and the targeted disruption of the *NDST1* gene impaired heart development in mice [46]. *NSDT1* ^f/f^/*2* ^null^/*3* ^null^ mice with defective decidualization resulted in female infertility [47]. It has been reported that *RSP14* is a key gene in early embryonic development [48]. Embryonic stem cells heterozygous for the *RSP14* gene showed defects in embryoid body differentiation [49]. For the GL trait, both GWASs based on chip data and imputed data identified genome-wide significant SNPs. Based on KEGG and GO analyses, we annotated a total of 13 candidate genes, mainly related to the ovarian steroidogenesis pathway and embryo development process. Among these, *ADCY5* was identified as being associated with seasonal estrus in Sunite sheep [50], egg production in white Muscovy ducks [51], and fertility in cows [52], while in human GWAS, *ADCY5* was found to be associated with gestational duration [53]. Interestingly, it has been reported that *ADCY5* is associated with fetal growth and birth weight [54]. However, the ADCY5 gene has not been studied in pig reproduction, and we speculate that this gene may be a key gene in the influence of reproductive performance in pigs. Unfortunately, no potential SNPs were identified for the NW trait, probably due to the small size of the population and the high number of missing phenotypic data points. Overall, our results identify a number of new key candidate genes and loci associated with reproductive traits in large white pigs, but further studies are needed to confirm the functions of these genes.

## 5. Conclusions

In this study, the genetic parameters of TNB, NBA, LBW, GL, and NW in Yorkshire pigs were estimated using a repeatability model. These traits are low-heritability traits. There were strong positive correlations between TNB, NBA, LBW, and NW, excluding the GL trait, which was weakly negatively correlated with them. GWASs based on chip data and imputed data were performed for five reproductive traits in Yorkshire pigs. Finally, combining the results of GWAS and bioinformatics annotation analysis, *SMAD4*, *RPS6KA2*, *CAMK2A*, *NDST1*, and *ADCY5* were identified as novel genes, and some of them have not been studied in livestock, so they may be key candidate genes affecting reproductive traits in pigs. The results of this study highlight some new major genes regulating reproductive traits in pigs and can benefit genome selection for pig genetic breeding.

## Figures and Tables

**Table 1:** Descriptive statistics for five reproductive traits.

Traits ^1^	N-obs ^2^	Mean	S.D.	CV ^3^ (%)	Min Value	Max Value
TNB	10,088	9.93	2.54	25.6	3	18
NBA	9862	9.25	2.53	27.2	3	17
LBW	9897	12.47	3.72	29.8	2.4	30
GL	10,193	114.65	1.49	1.3	105	127
NW	5857	8.75	2.46	28.1	2	17

^1^ TNB: total number born; NBA: number born alive; LBW: litter birth weight; GL: gestation length; NW: number of weaned. ^2^ N-obs: number of observations. ^3^ CV: coefficient of variation.

**Table 2:** Estimates of variance components and genetic parameters for five reproductive traits.

Traits ^1^	$\sigma_{a}^{2}$ ^2^	$\sigma_{pe}^{2}$ ^3^	$\sigma_{e}^{2}$ ^4^	h^2^	SE
TNB	0.2687	0.5289	5.2803	0.0442	0.0011
NBA	0.2700	0.5531	5.2856	0.0442	0.0012
LBW	0.6008	1.1104	10.9099	0.0476	0.0025
GL	0.3313	0.1765	1.6008	0.1571	0.0009
NW	0.4052	0.1744	4.9922	0.0727	0.0021

^1^ TNB: total number born; NBA: number born alive; LBW: litter birth weight; GL: gestation length; NW: number of weaned. ^2^$\;\sigma_{a}^{2}$: additive genetic variance. ^3^ $\sigma_{pe}^{2}$: permanent environmental effect variance. ^4^ $\sigma_{e}^{2}$: residual effect variance.

**Table 3:** Genetic correlations between five reproductive traits.

Traits ^1^	TNB	NBA	LBW	GL	NW
TNB		0.985 (0.001)	0.886 (0.003)	−0.235 (0.010)	0.751 (0.005)
NBA			0.945 (0.001)	−0.188 (0.010)	0.850 (0.003)
LBW				−0.120 (0.015)	0.934 (0.002)
GL					−0.176 (0.011)
NW					

^1^ TNB: total number born; NBA: number born alive; LBW: litter birth weight; GL: gestation length; NW: number of weaned. SEs of estimates are in parentheses.

**Table 4:** The significant SNPs in the genome for the gestation length (GL) trait using chip data in pigs.

Traits ^1^	SNP ^2^	Chr ^3^	Position	* p * -Value	Candidate Gene
GL	13:150210534	13	150210534	2.13 × 10^−7^	* DPPA4 * , *DPPA2*
	13:156135228	13	156135228	2.24 × 10^−7^	
	13:156180521	13	156180521	6.75 × 10^−7^	

^1^ GL: gestation length; ^2^ SNP: single-nucleotide polymorphism; ^3^ Chr: chromosome.

**Table 5:** The significant SNPs in the genome with the total number born (TNB) and gestation length (GL) traits using data imputed with PHARP.

Traits ^1^	SNP ^2^	Chr ^3^	Position	*p*-Value	Candidate Genes
TNB	6:77501624	6	77501624	6.07 × 10^−36^	*MRTO4, TAS1R2, PAX7, CAPZB, UBR4*
	6:77296986	6	77296986	1.36 × 10^−27^	*MRTO4, TAS1R2, PAX7, CAPZB, UBR4*
	12:10032955	12	10032955	1.16 × 10^−12^	*KCNJ2*
	13:51852849	13	51852849	7.91 × 10^−7^	*MITF*
	6:77325166	6	77325166	1.30 × 10^−11^	*MRTO4, TAS1R2, PAX7, CAPZB, UBR4*
	6:77330464	6	77330464	1.30 × 10^−11^	*MRTO4, TAS1R2, PAX7, CAPZB, UBR4*
	6:77458051	6	77458051	1.30 × 10^−11^	*MRTO4, TAS1R2, PAX7, CAPZB, UBR4*
	6:77462853	6	77462853	1.30 × 10^−11^	*MRTO4, TAS1R2, PAX7, CAPZB, UBR4*
	6:77480978	6	77480978	1.30 × 10^−11^	*MRTO4, TAS1R2, PAX7, CAPZB, UBR4*
	9:105582098	9	105582098	2.14 × 10^−11^	-
	6:77352307	6	77352307	8.67 × 10^−11^	*MRTO4, TAS1R2, PAX7, CAPZB, UBR4*
	6:77354514	6	77354514	8.67 × 10^−11^	*MRTO4, TAS1R2, PAX7, CAPZB, UBR4*
	6:77364237	6	77364237	8.67 × 10^−11^	*MRTO4, TAS1R2, PAX7, CAPZB, UBR4*
	6:77473320	6	77473320	8.67 × 10^−11^	*MRTO4, TAS1R2, PAX7, CAPZB, UBR4*
	6:77335250	6	77335250	4.47 × 10^−10^	*MRTO4, TAS1R2, PAX7, CAPZB, UBR4*
	6:77342385	6	77342385	9.30 × 10^−10^	*MRTO4, TAS1R2, PAX7, CAPZB, UBR4*
	6:77500110	6	77500110	9.30 × 10^−10^	*MRTO4, TAS1R2, PAX7, CAPZB, UBR4*
	6:77506794	6	77506794	9.30 × 10^−10^	*MRTO4, TAS1R2, PAX7, CAPZB, UBR4*
	6:77551399	6	77551399	9.30 × 10^−10^	*MRTO4, TAS1R2, PAX7, CAPZB, UBR4*
	6:77401218	6	77401218	9.30 × 10^−10^	*MRTO4, TAS1R2, PAX7, CAPZB, UBR4*
	2:41234740	2	41234740	1.89 × 10^−8^	*LDHA, LDHC, ABCC8*
	10:67101509	10	67101509	3.72 × 10^−8^	*PFKP*
GL	13:139111128	13	139111128	1.25 × 10^−8^	*ARGFX*
	13:141726529	13	141726529	1.96 × 10^−8^	*IGSF11*
	13:141659895	13	141659895	3.47 × 10^−8^	*IGSF11*

^1^ TNB: total number born; GL: gestation length; ^2^ SNP: single-nucleotide polymorphism; ^3^ Chr: chromosome.

**Table 6:** The significant SNPs in the genome for the total number born (TNB) and gestation length (GL) traits using data imputed with SWIM.

Traits ^1^	SNP ^2^	Chr ^3^	Position	*p*-Value	Candidate Gene
TNB	12:56839134	12	56839134	3.94 × 10^−20^	*MYOCD*
	12:56840928	12	56840928	3.09 × 10^−19^	*MYOCD*
	13:202985373	13	202985373	2..91 × 10^−12^	*HMGN1*
	11:43367981	11	43367981	2.78 × 10^−10^	*DACH1*
	11:60300851	11	60300851	3.06 × 10^−9^	*GPC5*
	10:3913625	10	3913625	3.82 × 10^−9^	-
	1:2245988	1	2245988	1.61 × 10^−8^	*RPS6KA2*
	11:60226963	11	60226963	2.43 × 10^−8^	*-*
NBA	8:27377546	8	27377546	3.44 × 10^−8^	*ARAP2*
	2:151525142	2	151525142	4.56 × 10^−8^	*RPS14, NDST1, CAMK2A*
	2:151616302	2	151616302	4.56 × 10^−8^	*CAMK2A, SYNPO, NDST1*
LBW	2:151525142	2	151525142	9.82 × 10^−17^	*RPS14, NDST1, CAMK2A*
	2:151635734	2	151635734	1.01 × 10^−14^	*RPS14, NDST1, CAMK2A*
	3:14559878	3	14559878	4.05 × 10^−10^	*AUTS2*
	3:2296285	3	2296285	3.29 × 10^−9^	*CARD11*
	6:30206625	6	30206625	4.60 × 10^−9^	*IRX6*
	8:27377546	8	27377546	2.24 × 10^−8^	*ARAP2*
	17:13034535	17	13034535	2.64 × 10^−8^	*PSD3*
GL	10:1796697	10	1796697	1.12 × 10^−8^	*RGS18*
	10:1805679	10	1805679	1.12 × 10^−8^	*RGS18*
	10:1809838	10	1809838	1.12 × 10^−8^	*RGS18*
	10:1820524	10	1820524	1.12 × 10^−8^	*RGS18*
	10:1838406	10	1838406	1.12 × 10^−8^	*RGS18*
	10:1847106	10	1847106	1.12 × 10^−8^	*RGS18*
	10:1855846	10	1855846	1.12 × 10^−8^	*RGS18*
	10:1784012	10	1784012	1.12 × 10^−8^	*RGS18*
	10: 1801316	10	1801316	1.12 × 10^−8^	*RGS18*
	10:1816938	10	1816938	1.12 × 10^−8^	*RGS18*
	10:1824028	10	1824028	1.12 × 10^−8^	*RGS18*
	10:1853098	10	1853098	1.12 × 10^−8^	*RGS18*
	10:1898784	10	1898784	1.12 × 10^−8^	*RGS18*
	10:1977819	10	1977819	1.12 × 10^−8^	*RGS18*
	10:1990160	10	1990160	1.12 × 10^−8^	*RGS18*
	10:2000211	10	2000211	1.12 × 10^−8^	*RGS18*
	10:1711812	10	1711812	1.68 × 10^−8^	*RGS18*
	10:1699892	10	1699892	1.96 × 10^−8^	*RGS18*
	10:1516875	10	1516875	2.38 × 10^−8^	*RGS18*
	10:1719667	10	1719667	2.63 × 10^−8^	*RGS18*
	10:1722698	10	1722698	2.63 × 10^−8^	*RGS18*
	10:1549545	10	1549545	3.75 × 10^−8^	*RGS18*
	10:1768905	10	1768905	4.79 × 10^−8^	*RGS18*
	10:1772064	10	1772064	4.79 × 10^−8^	*RGS18*

^1^ TNB: total number born; GL: gestation length; ^2^ SNP: single-nucleotide polymorphism; ^3^ Chr: chromosome.

**Table 7:** Significant KEGG pathways and GO terms associated with productive traits in pigs (*p* < 0.05).

Traits ^1^	Term	Database ^2^	ID	Gene Names
TNB	Glycolysis/gluconeogenesis	KEGG PATHWAY	ssc00010	*LDHC*|*LDHA*|*PFKP*
	TGF-β signaling pathway	KEGG PATHWAY	ssc04350	*SMAD4*|*NBL1*
	Oxytocin signaling pathway	KEGG PATHWAY	ssc04921	*KCNJ2*|*RYR2*
	uterus development	Gene Ontology	GO:0060065	*SMAD4*
	oocyte maturation	Gene Ontology	GO:0001556	*RPS6KA2*
NBA	Calcium signaling pathway	KEGG PATHWAY	ssc04020	*PDGFRB|ADRA1B|CAMK2A*
	GnRH signaling pathway	KEGG PATHWAY	ssc04912	*MMP2|CAMK2A*
	Embryonic organ development	Gene Ontology	GO:0048568	*PDGFRB*
LBW	MAPK signaling pathway	KEGG PATHWAY	ssc04010	*PDGFRB|GNA12|CSF1R*
	PPAR signaling pathway	KEGG PATHWAY	ssc03320	*FABP4|FABP5*
	Regulation of actin cytoskeleton	KEGG PATHWAY	ssc04810	*PDGFRB|GNA12*
	In utero embryonic development	Gene Ontology	GO:0001701	*PDGFRB|GNA12*
	Hormone receptor binding	Gene Ontology	GO:0051427	*FABP4*
	cell development	Gene Ontology	GO:0048468	*IRX5|IRX6*
GL	mTOR signaling pathway	KEGG PATHWAY	ssc04150	*ATP6V1C2|ATP6V1A|GSK3B*
	Ovarian steroidogenesis	KEGG PATHWAY	ssc04913	*ADCY5*
	Prolactin signaling pathway	KEGG PATHWAY	ssc04917	*GSK3B*
	Embryo development	Gene Ontology	GO:0009790	*DLX4|DLX3*
	Regulation of G protein-coupled receptor signaling pathway	Gene Ontology	GO:0008277	*RGS18*

^1^ TNB: total number born; NBA: number born alive; LBW: litter birth weight; GL: gestation length; NW: number of weaned. ^2^ KEGG: Kyoto Encyclopedia of Genes and Genomes.

## Data Availability

Data are available from the corresponding author on reasonable request.

## Supplementary Materials

The following supporting information can be downloaded at https://www.mdpi.com/article/10.3390/genes14040861/s1, Table S1. The genomic inflation factor (λ) for each GWAS using chip and imputation data of pigs. Table S2. Genes within 0.5 Mb of suggestive significant SNPs identified by GWAS based on chip data for reproductive traits. Table S3. Genes within 0.5 Mb of suggestive significant SNPs identified by GWAS based on the PHARP imputation data for reproductive traits. Table S4. Genes within 0.5 Mb of suggestive significant SNPs identified by GWAS based on SWIM imputation data for reproductive traits.
