# Supplementary material for: Genome-Wide Association Study on Reproductive Traits Using Imputation-Based Whole-Genome Sequence Data in Yorkshire Pigs
Source: Genes (Basel). 2023 Apr 2;14(4):861. doi: 10.3390/genes14040861 (PMC10137786; doi:10.3390/genes14040861)
Supplement: Supplementary file 1 [file genes-14-00861-s001.zip › Supplementary Table/Supplementary Table S1.pdf]

Supplementary Table S1. The genomic inflation factor (λ) for each GWAS using chip and imputation data in pigs.

Traits	TNB	NBA	LBW	GL	NW
λ (chip)	1.276	1.219	1.022	1.079	0.978
λ (PHARP)	0.964	1.095	1.012	1.030	0.976
λ (SWIM)	0.889	1.132	1.019	1.002	0.957
